# Characterization of the transcriptional and metabolic responses of pediatric high grade gliomas to mTOR-HIF-1α axis inhibition

**DOI:** 10.18632/oncotarget.16500

**Published:** 2017-03-23

**Authors:** Aurélia Nguyen, François Marie Moussallieh, Alan Mackay, A. Ercument Cicek, Andres Coca, Marie Pierre Chenard, Noelle Weingertner, Benoit Lhermitte, Eric Letouzé, Eric Guérin, Erwan Pencreach, Sarah Jannier, Dominique Guenot, Izzie Jacques Namer, Chris Jones, Natacha Entz-Werlé

**Affiliations:** ^1^ Laboratory EA 3430, Progression Tumorale et Micro-Environnement, Approches Translationnelles et Epidémiologie, University of Strasbourg, Strasbourg, France; ^2^ Department of Nuclear Medicine, University Hospital of Strasbourg, Strasbourg, France; ^3^ Institute of Cancer Research, Sutton, Surrey, United Kingdom; ^4^ Computational Biology Department, Carnegie Mellon University, Pittsburgh, PA, USA; ^5^ Computer Engineering Department, Bilkent University, Cankaya, Ankara, Turkey; ^6^ Department of Neurosurgery, University Hospital of Strasbourg, Strasbourg, France; ^7^ Department of Pathology, University Hospital of Strasbourg, Strasbourg, France; ^8^ Centre de Ressources Biologiques, University Hospital of Strasbourg, Strasbourg, France; ^9^ Programme Cartes d'Identité des Tumeurs, Ligue Nationale Contre Le Cancer, Paris, France; ^10^ Department of Pediatric Onco-hematology, University Hospital of Strasbourg, Strasbourg, France

**Keywords:** high grade glioma, pediatric, HIF1alpha, mTor, targets

## Abstract

Pediatric high grade glioma (pHGGs), including sus-tentorial and diffuse intrinsic pontine gliomas, are known to have a very dismal prognosis. For instance, even an increased knowledge on molecular biology driving this brain tumor entity, there is no treatment able to cure those patients. Therefore, we were focusing on a translational pathway able to increase the cell resistance to treatment and to reprogram metabolically tumor cells, which are, then, adapting easily to a hypoxic microenvironment. To establish, the crucial role of the hypoxic pathways in pHGGs, we, first, assessed their protein and transcriptomic deregulations in a pediatric cohort of pHGGs and in pHGG's cell lines, cultured in both normoxic and hypoxic conditions. Secondly, based on the concept of a bi-therapy targeting in pHGGs mTORC1 (rapamycin) and HIF-1α (irinotecan), we hypothesized that the balanced expressions between RAS/ERK, PI3K/AKT and HIF-1α/HIF-2α/MYC proteins or genes may provide a modulation of the cell response to this double targeting. Finally, we could evidence three protein, genomic and metabolomic profiles of response to rapamycin combined with irinotecan. The pattern of highly sensitive cells to mTOR/HIF-1α targeting was linked to a MYC/ERK/HIF-1α over-expression and the cell resistance to a major hyper-expression of HIF-2α.

## INTRODUCTION

Pediatric high grade gliomas (pHGGs), including diffuse intrinsic pontine gliomas (DIPGs), despite their low incidence, are the leading cause of mortality in pediatric oncology. Most pHGGs, localized in midline structures (thalamus and pons), are resistant to standard chemotherapy and harbor specific genomic alterations [1]. Studies have begun to unravel new cancer pathways specific to pediatric gliomagenesis with the discovery of frequent mutations of histones in those pHGGs and their impact on prognosis [2–4]. Cooperative international efforts are aimed at developing new prognostic and predictive biomarkers, underlying specific biology of these childhood malignant gliomas. Among those, hypoxic features seem to be good candidates and a translational way to target the pHGGs not depending on their specific molecular rearrangements and epigenetic deregulation. In fact, the necrosis resulting from severe intra-tumor hypoxia and the aberrant microvasculature, usually associated with it, are common radiological and histological adverse prognostic criteria in high grade gliomas [5]. Intra-tumor hypoxia is known to favor angiogenesis, but also genetic instability through resistance to apoptosis and decreased DNA repair capacities, promoting a highly chemo- and radio-resistant phenotype to cancer cells [6–8]. As the tumor proliferates and grows, tumor cells have to adapt to high anabolic requirements and to extreme environmental conditions due to the incapacity of the tumor microvasculature to provide sufficient oxygen and nutrient supplies. This adaptation to hypoxia is sustained by a whole metabolic reprogramming of cancer cells through the preferential use of alternative metabolic pathways, favoring glycolysis (the “Warburg” phenotype), but also glutaminolysis or de novo lipogenesis [8–11].

Cancer metabolic reprogramming is now considered as a hallmark of cancer and is largely driven by oncogenic and tumor suppressor gene deregulations, involving notably *TP53*, *AMPK*, *AKT*, *mTOR*, *HIF-1α*, *HIF-2α* and/or *MYC* [12–16]. The glycolytic switch of tumor cells is mainly induced by the accumulation of HIF (Hypoxia-inducible Factor) proteins. HIFs form heterodimers, whose α subunit is stabilized under hypoxia through the inhibition of the oxygen-dependent hydroxylases (PHDs) and its subsequent degradation by the proteasome. It can also be stabilized in normoxic conditions through different mechanisms of dysregulation at the transcriptional, translational and post-translational levels, leading to a pseudo-hypoxic phenotype [13–15]. HIF-1α and HIF-2α, the two most studied isoforms, activate numerous target genes. Noticeably, HIF-1α can activate the majority of genes involved in glycolysis, but, also, those involved in pH regulation, angiogenesis and metastatic progression [14, 15]. HIF-2α acts synergistically with HIF-1α to regulate many of these genes and its expression seems to be correlated with a more aggressive phenotype [16–18]. *TP53, MYC* and *AKT* cooperate with the HIFs to synergistically induce glycolysis, while *MYC* is the main regulator of glutaminolysis [19, 20]. Moreover, HIF-1α and HIF-2α have opposite effects on *MYC* or *TP53* stabilization. Therefore, the balanced expressions and interactions between those two isoforms are crucial for the understanding of the global cellular adaptation to hypoxia in cancers [19–21].

The other major regulator in hypoxia is mTOR. When mTOR is hyperactivated, it promotes HIF-1α protein activation, counteracting its degradation in normoxic conditions, leading to pseudo-hypoxia. Essential for tumor growth and survival, mTOR is also a sensor of nutrient level, favoring lipogenesis and inhibiting autophagy [21–23]. mTOR interacts with different protein partners, forming two distinct multiprotein complexes called mTORC1 (comprising RAPTOR and PRAS40) and mTORC2 (comprising RICTOR and PROTOR). mTORC1 is responsible for the downstream activation through its effects on S6K1 and 4EBP1, while mTORC2 is responsible for the complete activation of AKT and is interacting with HIF-2. S6K1 is a serine/threonine kinase that phosphorylates the S6 protein of the 40S ribosomal subunit (phosphorylated S6 ribosomal protein (phospho-S6RP or p-S6RP), which represent a specific surrogate of mTORC1 activation [24]. mTORC1 is positively regulated by the activation of PI3K/AKT and RAS/MAPK signaling pathways and negatively regulated by PTEN and AMPK. Under hypoxia, mTORC1 is also inhibited by REDD1 and BNIP3, themselves induced by HIF-1α and tightly regulated through a negative inhibitory feedback loop via S6K1. The upstream degradation of IRS1 and the inactivation of RICTOR/mTORC2 was also induced by S6K1 activation [24, 25]. mTOR inhibitors have shown limited efficacy in cancers, when used in monotherapy, partly due to the reactivation of AKT signaling pathway. This reactivation is mainly the consequence of the feedback loop induced by the residual HIF-1α expression and the balance between mTORC1 and mTORC2 [26–27].

The RAS-PI3K-mTOR pathway is altered in around 25% of pHGGs, while *PIK3CA/PIK3R1* and *TP53* are mutated in less than 40% [3, 28]. However, AKT and ERK have been reported to be highly activated at the protein level in those pediatric brain cancers, while PTEN expression is lost in less than 50% of them [1–3, 28, 29]. Others biomarkers, like *MYC* or *MYCN* amplifications, occurring at lower frequency in pHGGs, were reported associated with histone mutations, especially in diffuse intrapontine gliomas (DIPGs) and in histone G34V mutated pHGGs [28–34]. In terms of hypoxia, pHGGs display the classical characteristics of microvascular proliferation and/or necrosis, associated with intra-tumor hypoxia, although HIF-1 or HIF-2 over-expressions have not been specifically studied, but involvement only suggested. In those highly resistant tumors to chemotherapy and radiotherapy, a strategy of bi-therapy consisting of inhibiting both mTORC1 by rapamycin and HIF-1α by irinotecan might be efficient by completely abolishing HIF-1α accumulation [35, 36] and was evaluated in a pediatric phase I on malignant refractory tumors (ClinicalTrials.gov Identifier: NCT01282697, results are currently finalized).

To establish, the crucial role of mTOR/HIF-1α axis in pHGGs, we, first, assessed its protein and transcriptomic deregulations in a pediatric cohort of 26 pHGGs and in pHGG cell lines, cultured in both normoxic and hypoxic conditions. Secondly, based on the concept of a bi-therapy targeting mTORC1 and HIF-1α in pHGGs, we hypothesized that the distinctive genomic alterations of RAS/ERK and PI3K/AKT upstream signals and HIF-1α/HIF-2α/MYC relative expressions would differentially modulate the response to this double inhibition and impact on specific metabolic changes. The final objective of this preclinical study was to determine the predictive metabolomic, transcriptomic and protein profiles of response to this therapeutic combination in pHGG *in vitro* models.

## RESULTS

### mTOR/HIF pathway is highly activated in pHGGs samples, which can be grouped in 4 different hypoxia profiles

**Figure 1:**
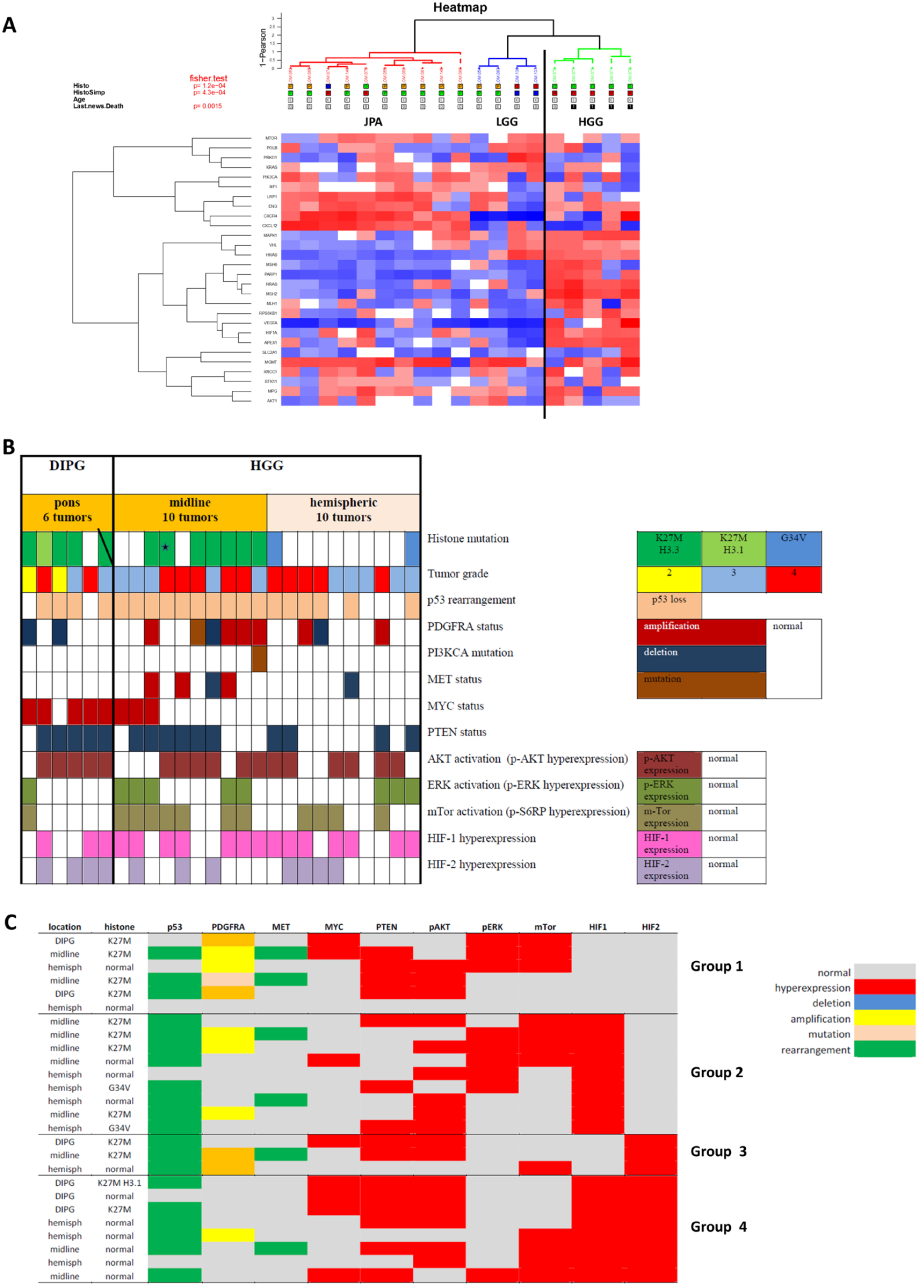
Status of the mTOR/HIF-1α/HIF-2α/MYC axis and their upstream and downstream signals in a patient cohort of 26 pHGGs **1A:** A transcriptomic analysis of hypoxic biomarkers in 5 pHGGs out of the 26 comparatively to 14 low grade gliomas (LGG) (the increased expressions are in red, the decreased expressions in blue and the normal one are in white). **1B:** The global description of over-expression, genomic alterations or mutations in the 26 pHGGs performed by several techniques and targeting mTor/HIF-1α axis deregulation (PI3KCA, MYC, PTEN, AKT, ERK, mTOR, HIF-1 and HIF-2) or pHGG/DIPG genomic characteristics. All normal results are in white boxes and each protein over-expression is in a corresponding color. **1C:** Based on the individual patient profiles, four subgroups could be identified regarding the balanced expression of the hypoxia surrogate markers HIF-1α and HIF-2α.

The first step of our study was to confirm the activation of mTOR-HIF-1/2α pathway in pHGGs. Therefore, we compared the RNA expression between 14 low grade gliomas and 5 pHGGs, included in the whole cohort of 26 patients described in the material section (Figure 1A). The 5 pHGGs were over-expressing predominantly and significantly *RAS, MAPK, AKT, VEGFA, VHL, HIF1* and *RPS6KB1* genes, involved in hypoxia pathways. Furthermore, in the whole cohort of 26 pHGGs (all data presented in Figure 1B), the immunohistochemical assessment (Supplementary Data 1) confirmed, as expected in the transcriptomic analyses, a frequent p-S6RP over-expression in 12/26 tumors and HIF-1α and HIF-2α over-expressions, respectively in 17 and 11 tumors out of 26. Eight patients had a concomitant HIF-1α and HIF-2α positive stainings. Only, three patients had an isolated HIF-2α hyper-expression. Four subgroups (Figure 1C) might be distinguished regarding to the hypoxia surrogate markers expressed within samples. The group 1 may be characterized by the complete absence of HIF-1α/HIF-2α expression, but for half of them a predominant Pi3K/PTEN/AKT activation is present and for the other half an ERK/mTOR expression. In the group 2, HIF-1α hyper-expression was linked in 5 patients to ERK/mTOR activation and in 4 cases to an isolated AKT hyper-expression. The presence of a HIF-2α hyper-expression in the groups 3 and 4 seems to be associated to a PTEN/AKT activation in 7 cases and to mTOR hyper-expression in 5 samples without any HIF-1α nor HIF-2α expressions.

In the 26 pHGGS (Figure 1B), the histone mutation was present in most of the DIPGs and the midline pHGGs. The G34V H3.3 mutation was rarer and only in hemispheric tumors (2 cases out of 10). There were no BRAF nor IDH1 mutations and only one tumor presents a PI3KCA mutation in exon 18. P53 rearrangement was frequently observed in 20 tumors. Frequent variations of copy number in *PDGFRA* and *MET* genes were found, whereas only one mutation of *PDGFRA* was diagnosed with Sanger sequencing in a midline pHGG.

The cell lines derived from patient tumors, TC35 and TC68, were harboring the same genomic and protein abnormalities than in the tumor themselves.

All those data were underlining the presence of an activated mTor/HIF-1α/HIF-2α pathway throughout DIPGs and pHGGs. To go further and to understand the functional role of hypoxia in pediatric high grade gliomas, we focused on those pathways in pHGGs’ cell cultures.

### Cell lines’ specific markers characterizing hypoxic conditions of culture

#### Hypoxia is clearly promoting mTOR signaling network activation in all cell lines (Figures 2A and 2B)

**Figure 2:**
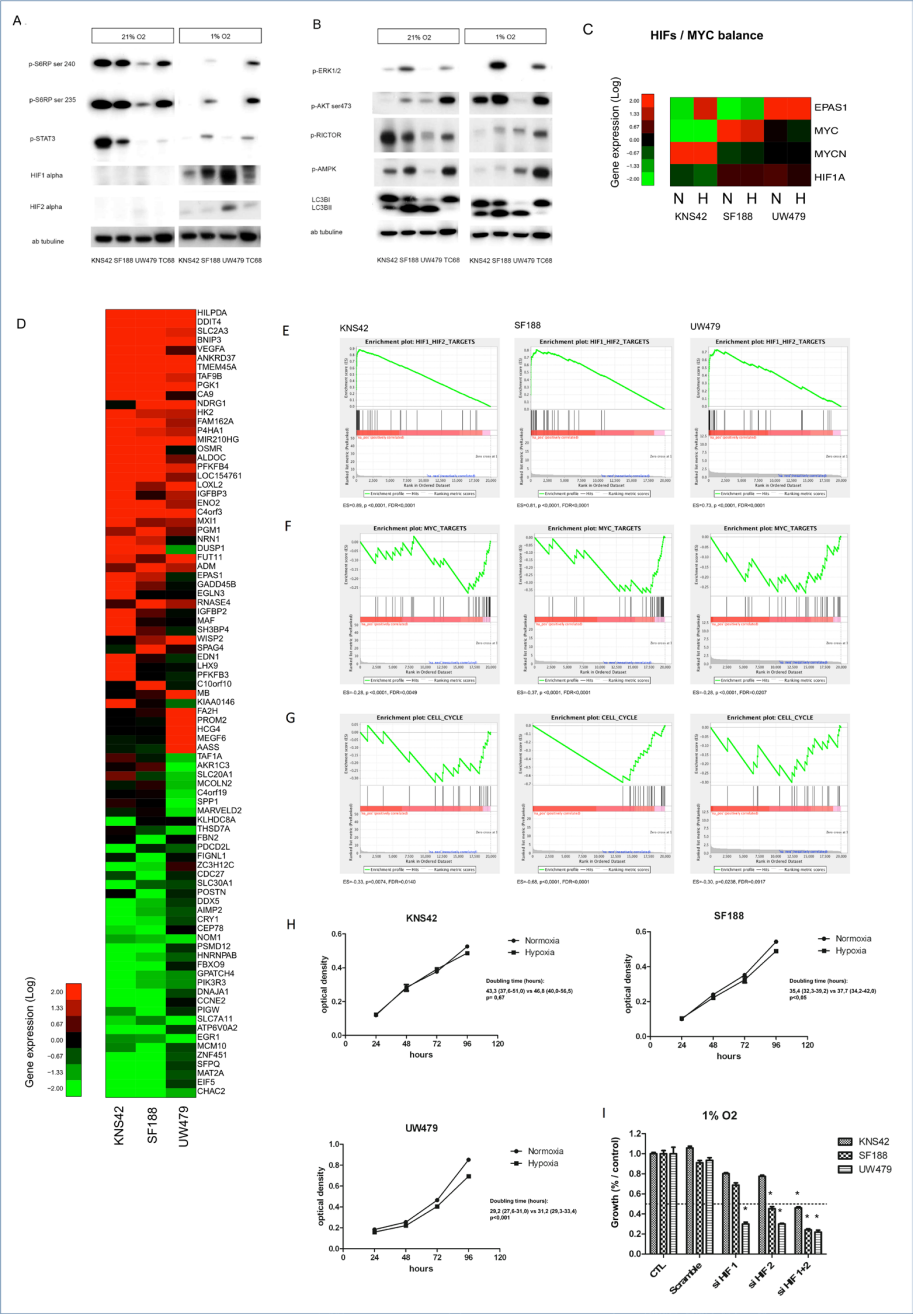
Impact of hypoxia (1% O2) on mTOR pathway activation, gene expression and proliferation in KNS42, SF188 and UW479 cell lines **Figure 2A and 2B:** Comparative immunoblotting analyses of mTOR pathway activation in KNS42, SF188, UW479 and the patient-derived cell line TC68 cultured in both normoxic and hypoxic conditions. **Figure 2C, 2D, 2E, 2F, 2G, 2H**: Comparative transcriptomic analyses. The relative expressions of *HIF-1/2α* and *MYC* are described in **Figure 2C**. Unsupervised analyses showing the top genes significantly upregulated or down-regulated in response to hypoxia (1% O2) comparatively to normoxia conditions are described in **Figure 2D**. Gene set enrichment analysis testing HIFs gene targets is shown in **Figure 2E**, MYC gene targets in **Figure 2F** and genes involved in cell cycle progression in **Figure 2G**. **Figure 2H** is presenting the impact of hypoxia on the proliferations of KNS42, SF188 and UW479, assessed by a colorimetric proliferation assay using crystal violet and the calculation of doubling time. In the **Figure 2I**, the effect on proliferation of siRNA transient transfection against HIF-1α, HIF-2α or both in SF188, KNS42 and UW479 cell lines in hypoxia. This effect was measured in triplicate and compared at 72 hours relatively to the control growth. *: p<0.05. ES: enrichment score; FDR: false discovery rate.

The impact of hypoxia on mTOR expression and its upstream PI3K-AKT and RAS-MAPK pathways was assessed, first, by immunoblot measures of their components in the cultures of the well-known and described cell lines, named KNS42, SF188 and UW479 [37]. In normoxic conditions of culture, KNS42, which is characterized by genomic gain at the locus of *PIK3CA*, an activation of IGFR1 and NMYC pathways and a histone H3.3 G34V mutation, presented the highest activation of mTORC1, as shown by the phosphorylation of mTORC1 downstream substrates (p-S6RP and STAT3) on Figure 2A. This mTOR activation was associated with an inhibition of mTORC2, shown by the inhibitory phosphorylation of RICTOR (p-RICTOR) and a low level of phospho-AKT (p-AKT) on Figure 2B. Under hypoxia, KNS42 presented a mTORC1 inhibition, which impacted on the inhibitory feedback loop of AKT with an increase of p-AKT and an activated mTORC2 network, showed by the dephosphorylation of RICTOR (Figure 2B). This feedback regulation was less prominent in the two other cell lines. Indeed, SF188 cell line, which is characterized by MYC amplification, *NF1* deletion and a transcriptomic hyper-expression of PDGFRA and KIT, showed an intermediate level of mTORC1 expression, but a higher level of phospho-ERK (p-ERK). For this cell line, a further activation of ERK in hypoxia was correlated with a less important inhibition of S6RP (Figure 2A and 2B). UW479, carrying an *IDH2* amplification associated with a hypermethylated phenotype, showed the lowest level of mTORC1 activation in normoxia. mTORC1 was also inhibited under hypoxia, but not associated with AKT nor ERK activations. Consistent with the role of mTOR inhibiting autophagy, the autophagy activity, reflected by the conversion of LC3B I in LC3B II, was especially present in UW479 in normoxia, where the lower level of mTORC1 induction was present. In hypoxia, the same autophagy levels than in normoxia were showed in the 3 cell lines (Figure 2B). TC68 had the same protein profile than SF188 cell line in both culture conditions except for autophagy induction, which was absent. TC35 was matching the results of TC68 and SF188 (data not shown). Thus, the mTORC1 pathway seems to be gradually activated throughout those cell lines from the lower level in UW479 to the higher level in KNS42 in normoxia, whereas hypoxia is inducing gradually the mTORC2 pathway with the lower level in KNS42 and the higher level in UW479.

#### HIF-1α is induced in hypoxic conditions in all cell lines, whereas HIF-2α hyper-expression is rarer

We compared the differential expression of HIF-1α and HIF-2α (or EPAS1) in all three cell lines (Figure 2A and 2C). In normoxia, no significant protein accumulation of HIF-1α or HIF-2α could be seen except a very slight increase in UW479 for HIF-2α protein (Figure 2A). The transcriptomic analyses confirmed the EPAS1/HIF2A over-expression in UW479 (Figure 2C). Under hypoxia, HIF-1α protein accumulation was inversely correlated to the level of mTOR activation, with the highest level in UW479 and the lowest HIF-1α expression in KNS42. HIF-2α protein was highly accumulated in UW479 during hypoxia. Those protein levels were correlated with their mRNA expression levels in hypoxia, as shown in RNA expression (Figure 2C). As expected, *MYCN* was strongly over-expressed in KNS42 and *MYC* in SF188. The transcriptomic analyses of the response to hypoxia comparatively to normoxia determined the top genes mostly upregulated (n=50) and down-regulated (n=38) (Figure 2D). Amongst those, 27 are well-described and are HIF-1α targets, which are upregulated only in hypoxia conditions, confirming the hypoxic induction of HIF-1α. The GSEA analyses confirmed the strong enrichment in HIF1/2 target gene expression in all three cell lines when hypoxia is present (p<0.0001). Consistent with the HIF-1α inhibition of MYC target genes in hypoxia, a significant down-regulated expression of MYC target genes, involved in cell cycle progression, was observed in the three cell lines (Figure 2E, 2F and 2G). As shown by the negative enrichment scores, there was a higher significance in SF188, where a spontaneous and known MYC amplification is present. Thus, the hypoxia is inducing HIF-1α expression in all cell lines, but, is clearly and significantly impacting on MYC target genes involved in cell cycle in SF188 and KNS42, where *MYC* or *NMYC* is spontaneously expressed in the standard condition of normoxia. HIF-2α is especially induced in UW479 cell line in absence of *MYC/NMYC* over-expression.

#### Cell proliferation is preserved even in hypoxia

Because of this negative effect on cell cycle gene expression, we looked at cells’ proliferation. KNS42 cells had no modification in their proliferation, whereas a slight inhibitory effect on the proliferation of SF188 and UW479 was observed (Figures 2H) at 72h, based on the analyses of doubling time (p<0.05). To determine the respective impact of HIF-1α and HIF-2α RNA expression on the proliferative capacities of those cell lines under hypoxia, a transient transfection was performed with siRNAs against HIF-1α, HIF-2α and the combination of both (Figure 2I). The inhibition of HIF-1α and/or HIF-2α contributed to a proliferation inhibition in UW479. A 75% decrease in case of HIF-1α or HIF-2α inhibition was observed and a higher significantly inhibition with the combination of both siRNAs was present (p<0.05). In KNS42 and SF188, the inhibition of HIF-1α had no significant anti-proliferative effect. The inhibition of HIF-2α led to a surprising and moderate effect in SF188 (50%, p<0.05), while a stronger effect in SF188 was observed with the combined inhibition leading to an 80% growth inhibition (p<0.05). This inhibitory effect in SF188 is predominantly observed with HIF-2α and efficient in hypoxia conditions, where MYC target genes are clearly and significantly inhibited by hypoxia itself and subsequent to HIF-1α induction (Figure 2F and 2G). Therefore, the unique inhibition of HIF-1α is probably increasing the MYC target gene expression and promoting a partial proliferative effect in SF188, whereas the combined inhibition is more decreasing the MYC target genes’ effects on cell proliferation.

In conclusion, cell proliferation seems to be preserved in hypoxia conditions

#### Hypoxia is contributing to specific metabolic switches according to the initial induced protein profiles

**Figure 3:**
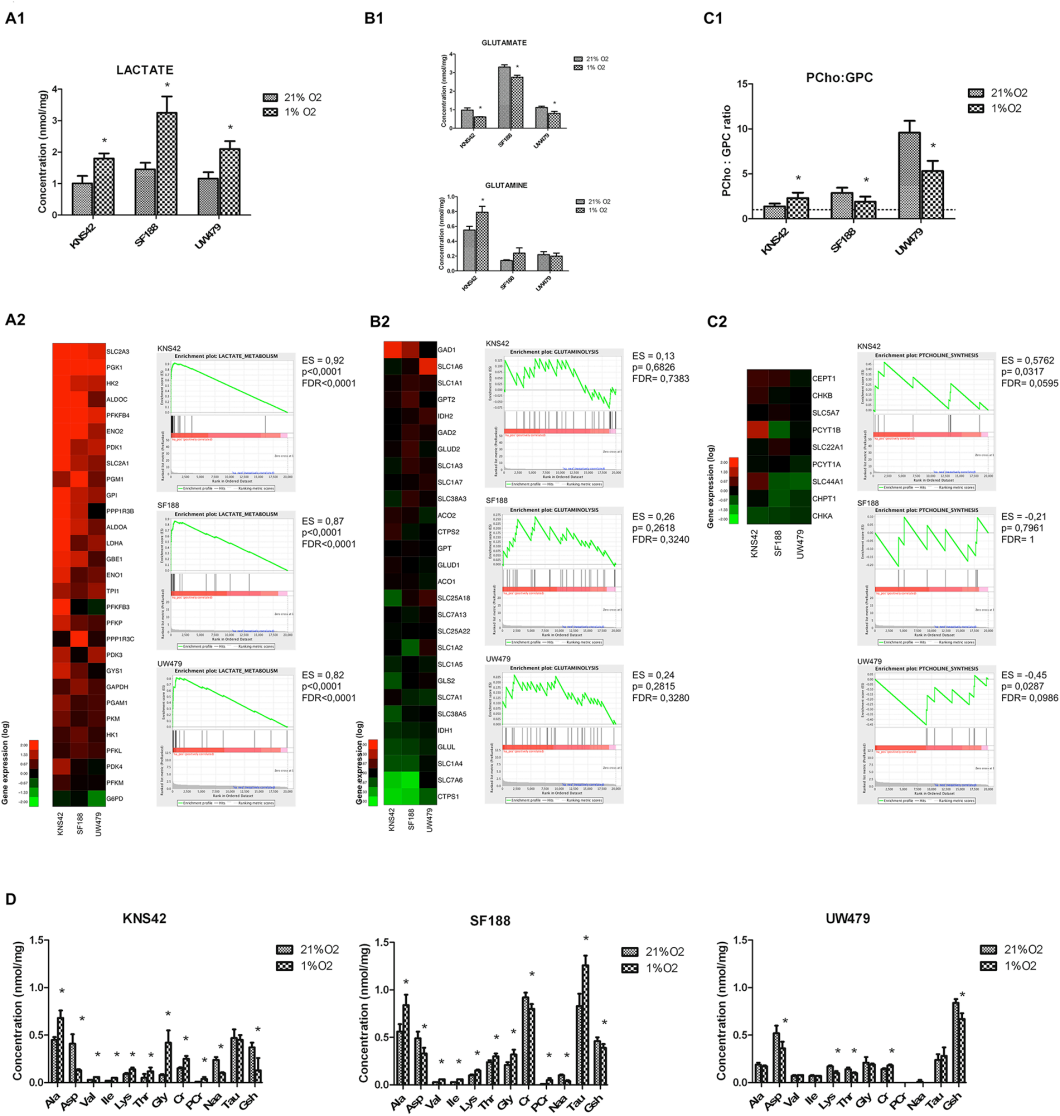
*In vitro* metabolomic analyses of KNS42, SF188 and UW479 cell lines cultured in hypoxia (1% O2) Each metabolic pathway was comparatively analyzed in terms of metabolite accumulation by HRMA NMR spectroscopy (concentrations expressed in nmol/mg) and in terms of gene expression by microarrays with GSEA analysis. Glycolysis was analyzed in terms of lactate accumulation and gene expression (**Figure 3A1, 3A2**), glutaminolysis in terms of glutamine and glutamate accumulations and gene expression (**Figure 3B1, 3B2**), the lipid synthesis and degradation pathway in terms of PCho:GPC ratio and gene expression (**Figure 3C1, 3C2**), the amino-acid, creatine and gluthatione (GSH) pathways in terms of metabolite accumulations (**Figure 3D**). Ala: alanine; Asp: aspartate; Val: valine; Ile: isoleucine; Lys: lysine; Thr: threonine; Gly: glycine; Cr: creatine; PCr: phospho-creatine; Naa: N-acetyl-aspartate; Tau: taurine; Gsh: gluthatione; PCho: phosphor-choline; GPC: glycerophosphocholine; PtCholine: phosphatidyl-choline.

In order to understand the changes in metabolic fluxes associated with hypoxia, the metabolomic profiles were compared to gene expression and to the protein profiles of KNS42, SF188 and UW479 (Figure 3). A significant accumulation of lactate was evidenced in all three cell lines in hypoxia, correlated with an up-regulation of the glycolysis genes at the mRNA level (Figure 3A1 and 3A2). SF188 was characterized by the highest accumulation of lactate in hypoxia, which was concordant with the accumulation of glutamate, associated with the highest basal up-regulation of glutaminolysis gene expressions (Figure 3B2). The metabolomic profile of SF188 in Figure 3D confirmed with the accumulation of glutamine, aspartate, alanine and glycine the glutaminolysis activation. The glycine, its metabolic derivatives (creatine and phosphocreatinine), alanine and taurine were also accumulated in SF188 and were the witnesses of a concomitant over-expression of the serine/glycine biosynthetic pathway genes (Figure 3C and 3D). In KNS42, the accumulation of glutamine, alanine, taurine and glycine reflected the concomitant activation of glutaminolysis and serinolysis in both conditions of culture, but, comparatively to SF188 cell line, the activation of serinolysis was predominant in hypoxia, as the glycine and glutamine levels were persistent and higher (Figure 3A2 and 3D). Lipid storage and triacyl-glycerol synthesis pathways were significantly induced by hypoxia in KNS42, where a concomitant up-regulation of lipogenesis regulators and the genes of lipid biosynthesis was present (Figure 3C2). The increase of the PCho:GPC (phosphocholine/glycerophosphocholine) ratio, reflecting an increase in phosphatidylcholine (PtCholine) synthesis (Figure 3D), confirmed the induced lipogenesis in KNS42. In UW479, in both conditions of oxygenation, the only major metabolic pathway was lipolysis, which was characterized by the PCho:GPC ratio decrease concomitantly with the induction of PtCholine degradation genes (Figure 3C and 3D) and the up-regulation of genes involved in lipolysis.

In conclusion, SF188, where MYC and mTORC1/p-ERK/HIF-1α were overexpressed, was characterized by a glycolysis and a glutaminolysis induction in hypoxia. For KNS42, where NMYC and mTORC1/p-AKT were overexpressed, a predominant glycolysis, serinolysis and lipogenesis induction was present. Finally, the UW479, mainly characterized by its spontaneous HIF-2α hyper-expression, had a major lipolysis induction in hypoxia conditions.

### Sensitivity to mTOR/HIF-1α inhibition correlates positively with AKT/PI3K activation and negatively with HIF-2α accumulation

#### Proliferation impact of those targeted treatments

**Figure 4:**
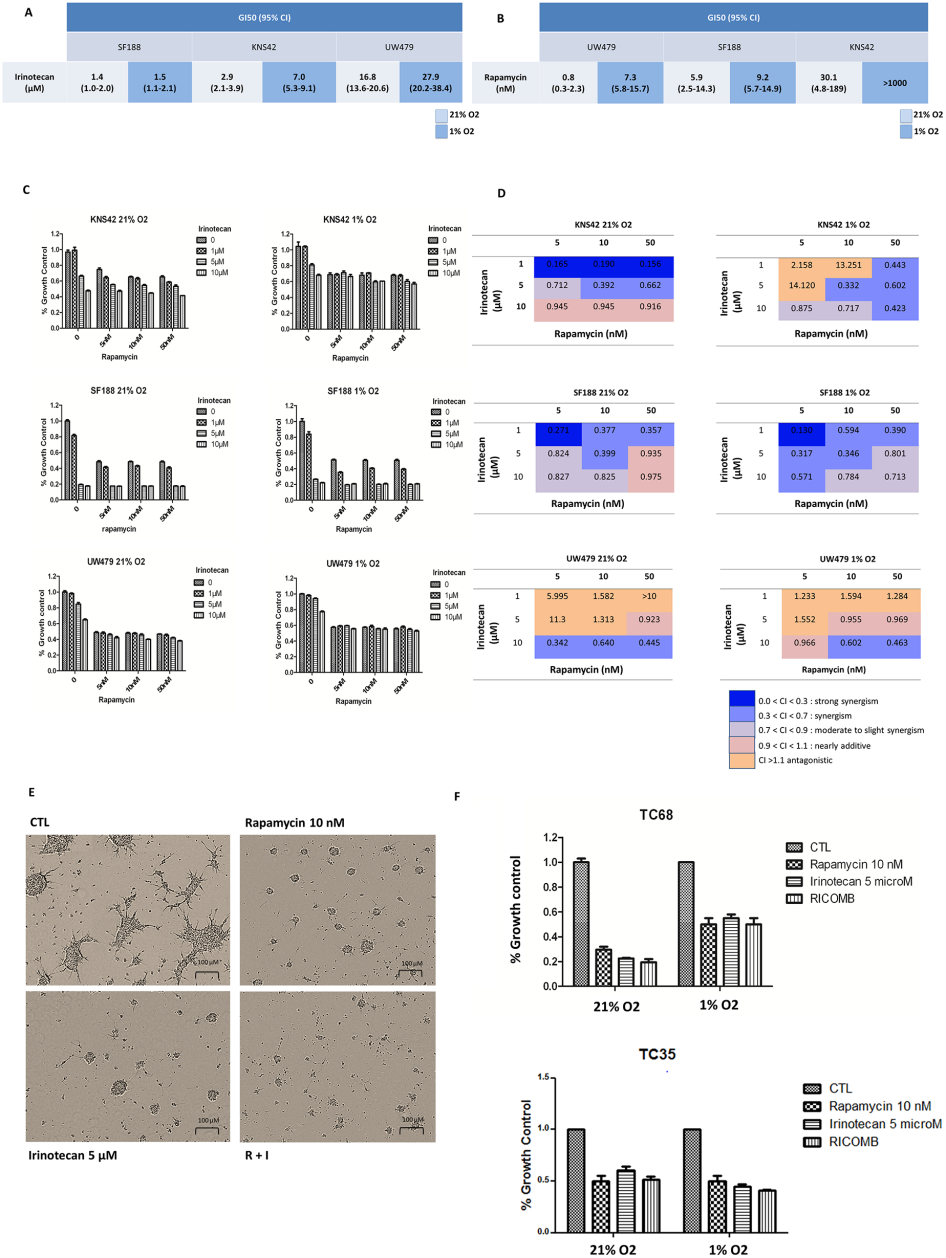
Efficacy of rapamycin, irinotecan and the combination of both in terms of anti-proliferative effect measured at 72 hours from treatment in normoxic and hypoxic conditions in the 3 cell lines and in TC68/TC35 This anti-proliferative effect was measured using a crystal violet colorimetric proliferation assay in the 3 cell lines and a trypan blue exclusion assay with automated counting of cells in TC68 and TC35. The 50% growth inhibition (GI50) values (95% CI) are reported for irinotecan in **Figure 4A** and for rapamycin in **Figure 4B**. Proliferation curves are reported in **Figure 4C** and combination indexes (CI) in **Figure 4D**. For TC68, the effect of drugs on neurosphere formation is described in **Figure 4E** (phase contrast microscopy) and the effect on proliferation in **Figure 4F** for TC68 and TC35.

The GI50 values are reported in Figure 4A for irinotecan and Figure 4B for rapamycin. They were obtained for small doses of rapamycin (ranging from 0.3 to 30.1 nM in normoxia and 5.8 to more than 1000 nM in hypoxia). However, the effect on proliferation was moderate in all three cell lines (Figure 4C). In normoxia, UW479, the most sensitive cell line to rapamycin, showed a maximal plateau effect of 50% from a dose of 1 nM. SF188, harboring intermediate sensitivity to rapamycin, showed the same response with a decrease rate of 50% from 5 nM, whereas KNS42, the most resistant, reached a maximal 50% growth inhibition for a dose of 100 nM of rapamycin. In hypoxia, the same effect (decrease of 50% of cell proliferation) was obtained for higher doses of rapamycin. UW479 was highly resistant to irinotecan in normoxia and hypoxia. With a GI50 around 1.5 μM in normoxia and hypoxia, SF188 was the most sensitive to irinotecan, while KNS42 showed a moderate response.

In order to assess the potential synergy of the combination, we calculated the combination index of both drugs with the Chou and Talalay method (rapamycin: 5, 10 and 50 nM and irinotecan: 1, 5 and 10 μM) (Figure 4D). A quite constant synergism in drug combinations was observed in SF188 cells, where the smallest doses of rapamycin and irinotecan (5 nM and 1 μM, respectively) in both normoxia and hypoxia were efficient. In KNS42, a clear synergy (CI<0.9) could be obtained in normoxia with the combination but only with the lower doses of irinotecan independently from rapamycin doses. The higher dose of irinotecan combined with rapamycin only showed an additive effect, whereas, in hypoxia, a synergism could be obtained by increasing the doses of rapamycin and irinotecan. Therefore, the optimal doses of those drugs in both oxygen conditions might be 10 nM of rapamycin and 5 μM of irinotecan in KNS42 cells. In UW479, overexpressing HIF-2α, a higher dose of irinotecan 10 μM with rapamycin from 10 nM was necessary to obtain a regular synergism in both culture conditions. So, the common doses, which might give in most of the cells (SF188 and KNS42) a “regular” synergism, were 10 nM of rapamycin and 5 μM of irinotecan. We considered the UW479 as the less sensitive cells or even the resistant cells to the treatment combination.

We tested those doses in both conditions in TC68 and TC35 (presenting K27M histone H3.3 mutations). TC68 cells, overexpressing concomitantly MYC, p-AKT and HIF-1α proteins (Figure 1B, the DIPG tumor with the bar), close to the protein profile of SF188 cells, were showing an equivalent sensitivity as SF188 cells. The cell viewing with Incucyte^©^ technology in TC68 showed a high decrease (automated calculations) of neuropsheres’ size with rapamycin, irinotecan and the combination (Figure 4E and 4F). TC35, overexpressing mTORC1 and p-AKT as in KNS42 cells (Figure 1B, the pHGG tumor with the star), presented an intermediate response in terms of growth control.

#### The bitherapy is clearly effective in SF188 cells considering protein responses (Figure 5)

**Figure 5:**
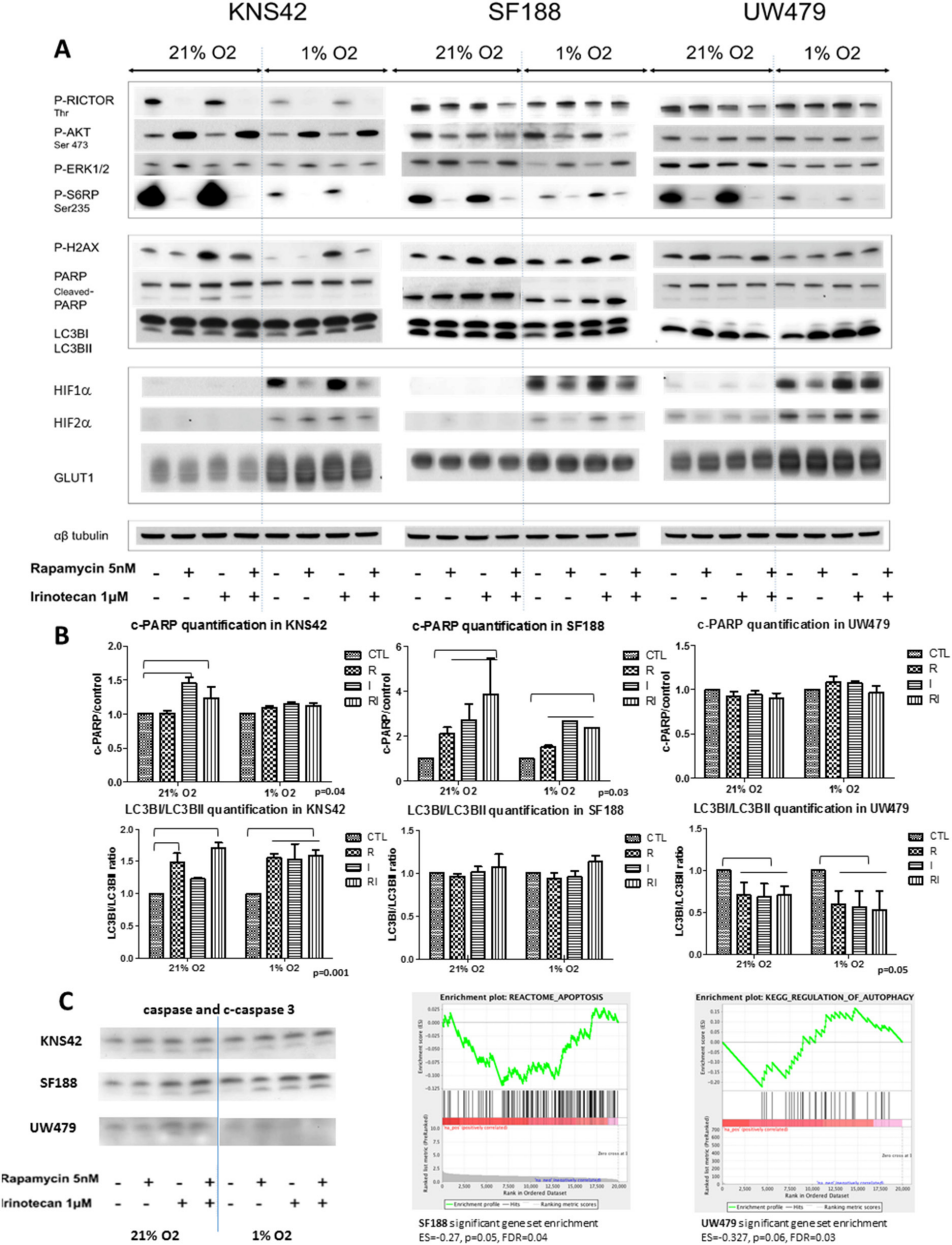
Cell effects of rapamycin, irinotecan and the combination and impact on the mTOR-HIF-1/2α pathway in KNS42, SF188 and UW479 in both normoxic and hypoxic conditions The immunoblotting analyses of the protein expressions are presented for the cleaved PARP, phospho-H2AX and LC3B as markers of apoptosis, double strand breaks and autophagy, respectively, and for the phosphorylated forms of RICTOR, AKT, ERK and S6RP as markers of mTOR pathway and HIF-1/2α protein accumulations in **Figure 5A**. The **Figure 5B** is illustrated the quantification of western blot analyses (c-PARP/control ratio and LC3BI/LC3BII ratio). The **Figure 5C** is presented cleaved-caspase 3 western blot analysesfor each cell line and the significant gene set enrichment analyses testing apoptosis genes in SF188 and autophagy pathway in UW479.

**Figure 6:**
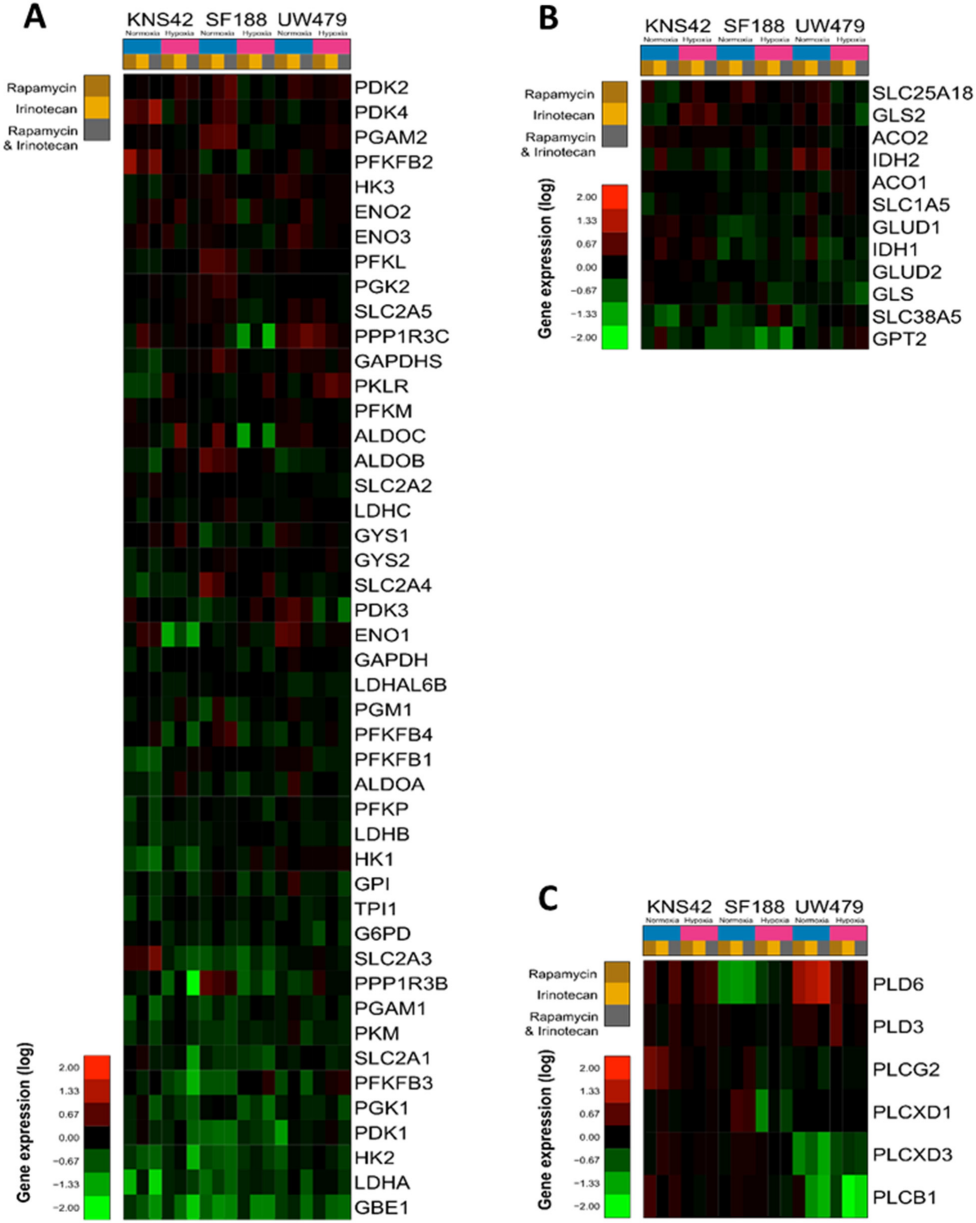
Effects of rapamycin, irinotecan and the combination of both on metabolism gene expression in KNS42, SF188 and UW479 The results of glycolysis pathway deregulation are presented in **Figure 6A**. The gene deregulations in the glutaminolysis pathway are described in **Figure 6B** and the effects on phosphatidyl-choline degradation pathway are showed in **Figure 6C**.

KNS42 response to rapamycin was characterized in normoxia and hypoxia by a strong activation of mTORC2 as shown by the phosphorylation of AKT with a concomitant dephosphorylation of RICTOR (Figure 5A). P-AKT was not induced in SF188 and UW479 and even slightly repressed. As expected, the downstream substrate of mTORC1, S6RP, was importantly repressed by rapamycin in all three cell lines. P-ERK was strongly induced by rapamycin in SF188 and particularly in hypoxia, while it was only slightly induced in KNS42 and not in UW479. P-AKT or p-ERK expressions were not influenced by irinotecan alone or in combination with rapamycin (Figure 5A). HIF-1α accumulation was strongly repressed under hypoxia by rapamycin in KNS42 and only partially in SF188 and UW479. HIF-2α was not repressed in KNS42 under hypoxia, but was repressed in SF188 and more slightly in UW479. Noticeably, there was no inhibition of HIF-1α nor HIF-2α protein accumulations in any of these cell lines by irinotecan monotherapy. In UW479, an increase of HIF1 and HIF2 accumulation could be observed with the combination of the 2 drugs. At the mRNA level, only, few HIF-1/2α target genes were significantly and differentially inhibited by rapamycin in KNS42 and SF188 (Figure 6A), while no significant down-regulation, for example in SLC2A3, PGK1 or PDK1 expressions, could be observed in UW479. The increase of HIF-1α/HIF-2α in UW479 cells with the bitherapy might explain the resistance of those cells to this mTOR/HIF-1α targeting. The major role of HIF-1α and HIF-2α in UW479 cells was already underlined in Figure 2I, where UW479 cells were dramatically sensitive in terms of cell proliferation to direct siRNA targeting of those proteins.

The cleaved-PARP, witness of apoptotic cells, was only present in KNS42, when treated with irinotecan in normoxic conditions, along with a concomitant increase of DNA double strand breaks (increase of phosphoH2X) and autophagy (presence of LC3BII) was increasing in case of rapamycin treatment in normoxia (Figure 5A and 5B). Treatment of SF188 cells showed apoptotic cells in all conditions of culture and treatment, confirming SF188 as the more sensitive cell line to mTOR/HIF-1α targeting. This expression of cleaved-PARP was completely matching phosphoH2AX expression and the cleaved-caspase protein expression, whereas autophagy was constant and not depending on the type of treatment nor culture conditions (Figure 5B and 5C). The GSEA analyses confirmed the apoptosis pathway involvement in SF188 (Figure 5C) and the constantly induced autophagy was confirmed at mRNA levels with the spontaneous increased expressions in all conditions and treatments of autophagy genes, like ULK2, ATG14 or ATG12 (Supplementary Data 2). UW479 cells seem, as already showed above, to be resistant to mTOR and HIF-1α targeting with a highly induced autophagy especially under hypoxia and without any visible apoptotic cells (Figure 5A, and 5C).

#### Resistance to rapamycin and/or irinotecan inhibition on cell metabolism was based on an induced lipolysis

**Figure 7:**
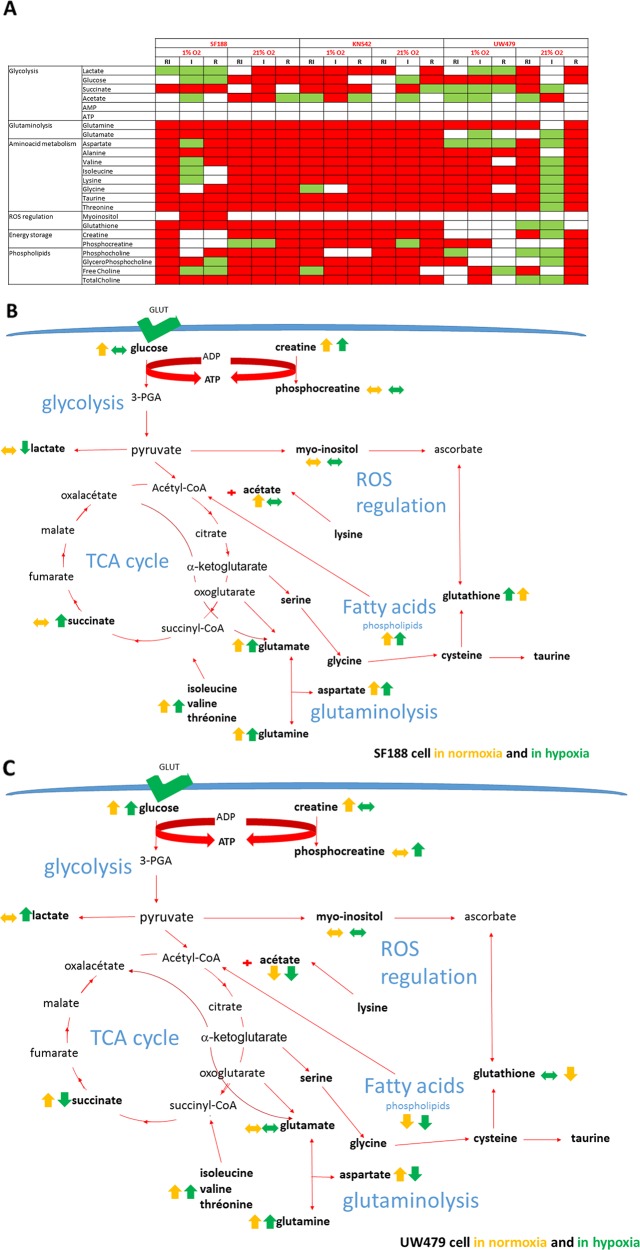
Effects of treatment on metabolite accumulation The results are expressed relatively to control and a significance (p<0.05) was present when compared cell lines and culture conditions. In **Figure 7A**, the changes are shown in red color when the metabolites are increasing, in green color when they are decreasing and in white when the expression was normal. In **Figures 7B** and **7C**, the cartoons are describing the metabolites and their pathway deregulation (yellow arrows in normoxic culture, green arrows for hypoxic conditions). The figure 7B is representing the specific metabolite deregulation in the more sensitive cells (SF188 cell line) and the figure 7C in the more resistant line (UW479 cell line).

The glycolytic pathways upregulated in all cell lines under hypoxia were poorly impacted with the targeting of mTOR/HIF-1α in the cells. In fact, the majority of glycolysis genes were not down-regulated, as well as the protein expression of GLUT1 (SLC2A1), a downstream target of HIF-1α (Figure 5A, 6A and 7A). The metabolomic analyses reveal no significant decrease in the glucose or lactate metabolites, when the cells were treated with rapamycin or irinotecan during normoxia or hypoxia (Figure 7A). An increased level of glucose was even present in the resistant UW479 cells in both conditions and with all treatments. In the most sensitive cells, SF188, the only significant glycolytic metabolite was an increased level of succinate. The phospho-creatine:creatine ratios increased when treated KNS42, whereas this ratio was intermediate in SF188 and decreased completely in UW479. So, the response to treatment in SF188 and in KNS42 seems to be an excess of glycolysis with an increase of phosphocreatine, even the ATP production seems not to be significantly increasing during metabolomic assessment. In parallel, the level of ROS regulation metabolites in Figure 7 was significantly increasing in responding cells to almost all drugs in both conditions and was not changing in the resistant UW479 line.

Nevertheless, glutaminolysis was strongly inhibited by rapamycin in SF188, as shown by the inversion of the glutamate/glutamine ratio (<0.25) and the down-regulation of glutaminolysis genes (Figure 6B and 7). Irinotecan monotherapy had an inhibitory effect on glutaminolysis in SF188 and, only, in normoxia. In SF188, the combination of both drugs led to a further glutaminolysis inhibition in both cell lines and in both culture conditions and was confirmed by the accumulation of aspartate, alanine, valine, isoleucine, lysine and glycine (Figure 7A). In KNS42, no inhibition was detected and, only, a moderate inhibition was underlined in UW479 cells with a slight inversion of the glutamate/glutamine ratio (0.69).

For the phospholipid pathway usually influenced by the modulation of mTOR, the decrease of the phosphocholine level was present in case of UW479 resistant cells to bitherapy. Correlating with these observations, some phospholipases at the origin of the generation of GPC were down-regulated at the mRNA level particularly in UW479 under hypoxia (Figure 6C) and confirmed the increase of the lipolytic pathways. On the contrary, the levels of both phosphocholine and GPC were increasing in SF188 cells to combined therapies with a probable inhibition of the residual lipolysis in the cells.

The Figure 7B and 7C are summarizing the metabolomic signatures observed after concomitant inhibition of mTor/HIF1 axis in the more sensitive cells, SF188, in both culture conditions and in the resistant UW479 cells. In fact, the more sensitive cells to the combination of rapamycin and irinotecan were defined with an excess of glycolysis and a high inhibition of glutaminolysis and lipolysis. In the resistant cells, UW479, the metabolic signature was characterized mainly by the absence of inhibition in the lipolytic pathways.

### The *in vivo* translation into patient samples showed comparable profiles in protein expression and culture

The immunohistochemical analyses in the 26 patients’ tumors screened different patient subgroups based on the AKT/ERK and HIF-1α/HIF-2α balances. In those analyses, we could isolate, as in UW479 cells, a small subgroup with an isolated HIF-2α over-expression. It represents a very few number of patients in the group 3 (Figure 1C), only 2 patients had those protein criteria. The SF188 profile is typically associating a concomitant p-ERK/MYC hyper-expression with a HIF-1α over-expression or normal expression, as in the groups 1 and 2 and in the group 4. Among the 8 MYC amplified tumors, there are 4 patients corresponding to the complete SF188 protein profile. In the same groups, KNS42 profile could be also outlined in 3 tumors, if we are combining the p-AKT hyper-expression plus mTor expression without any HIF-2α expression. Nevertheless, we might also have a less restricted protein profile based on the cell behavior after combined treatment, as the slide of the tumor is representing an unknown timepoint and spacepoint in the tumor itself. We could, then, base our tumor selection for sensitivity on the balance between p-AKT, p-ERK, MYC or mTor, which concomitantly or independently are modulating the sensitivity to rapamycin and irinotecan, and in the absence of an isolated HIF-2α over-expression. This sensitive group is, then, reaching one third of the tumors. To afford a partial confirmation of the drug sensitivity, in patient-derived cell lines, we also tested the double inhibition of mTOR and HIF-1α. In TC68, bearing *H3.3 (K27M*), *TP53* mutations and a similar protein profile to SF188, cells’ response was similar to SF188 response in both conditions and confirmed the therapeutic potential of this combination in sensitive pHGGs.

## DISCUSSION

As no real data on the role of hypoxia in pHGGs are published up to date, our first step in this work was to analyze tumor samples of pHGG and DIPG to screen if biomarkers involved in mTOR/ HIF-1α pathway were upregulated. Our data showed a major activation and/or over-expression of all markers justifying a further study in pHGGs cell lines testing the hypoxia conditions themselves and the concomitant inhibition of mTor and HIF-1α. Another argument to target such pathway was the fact that initiating tumor cells are often localized in hypoxic niches within tissues, usually restarting at relapse even after a curative treatment. Recent publications are considering those cells as the key in the tumor initiation and the growth maintenance of a number of pediatric brain tumors including the high grade gliomas, where hypoxia inducible factors (HIFs) seem to play key roles in those cells [38, 39].

In the cell study, we showed, first, that the dysregulation of MYC/MYCN and the mTOR/HIF pathway determined the responses of pHGG cell lines to hypoxia and their response to the pharmacological inhibition of mTOR and HIF-1α. As mTOR is known to be physiologically inhibited by hypoxia, the first question was to understand the benefit of a further inhibition by rapamycin in hypoxic tumors based on the oncogenic and metabolic adaptations adopted by the cells to survive and proliferate in those conditions.

In KNS42, the induction by hypoxia of AKT, HIF-1α and HIF-2α in conjunction with the basal *MYCN* over-expression lead to a complete metabolic reprogramming through the main activation of glycolysis, glutaminolysis and lipogenesis [40]. Compared to the other cell lines, KNS42 was the only one to induce the “*de novo*” lipogenesis pathway under hypoxia correlating with the best adaptation to hypoxia. The upregulation of the transcription factor SREBP-1, master regulator of lipogenesis sustained this specific induction in KNS42 RNA expression profiles. Although supposed to be under the positive control of mTORC1, several studies have highlighted the role of mTORC2 and AKT in activating lipogenesis through SREBP-1, linking this reactivation to the best metabolic adaptation to hypoxia for tumor cells [41–44]. The inhibition of mTORC1 and mTORC2 seem to impact on this metabolic adaptation and could help even in hypoxia to stop cell proliferation [45]. In comparison, AKT was not activated by hypoxia in SF188 and UW479, consistent with their down-regulation of SREBP-1 and lipogenesis genes.

SF188 showed, as already described, an important basal activation of glutaminolysis promoted by *MYC* amplification [46, 47]. In the cell lines using glutaminolysis, this convergence towards glutamate utilization is used to fuel the TCA cycle to produce energy and sustain lipogenesis [47]. The response of SF188 to hypoxia was characterized by a high activation of glycolysis and reductive glutaminolysis along with a moderate HIF-1α and a slight HIF-2α accumulation. Promoting proliferation and survival, a predominant p-ERK activation could be observed in SF188 and related to its *NF1* deletion.

In normoxia, the third cell line, UW479, was characterized by low levels of mTOR, ERK and AKT expressions, without MYC or MYCN dysregulation. It was also characterized by higher level of free amino acids, potentially resulting from its higher basal autophagic activity and is usually inducing mTor expression [48, 49]. Moreover, the lipases MAGL and LPL were over-expressed in UW479 and are well-known markers of aggressiveness in cancer [50]. Therefore, UW479 may rely on lipolysis and autophagy to sustain its energy demand and its adaptation to hypoxia, promoting this cell as a highly resistant one. In the absence of MYC or NMYC dysregulation, the very high accumulations of HIF-1α and HIF-2α observed in UW479 under hypoxia were correlated with a less predominant activation or addiction to glycolysis and glutaminolysis.

It is likely that of all these factors act synergistically in the promotion of the specific metabolic phenotype of each cell in normoxic and hypoxic conditions and may result from a subtle balance. Interestingly, HIF-1α and HIF-2α act in opposite ways to regulate cell cycle progression, by inhibiting (for HIF-1α) or promoting (for HIF-2α) MYC/MYCN stabilization or transcriptional activity [20, 21].

In KNS42 and SF188 in which MYC/MYCN was deregulated and HIF-2α really slightly expressed, most of the genes involved in cell cycle progression were down-regulated under hypoxia. Nevertheless, in UW479, in which HIF-2α was highly expressed, this latter was not able to promote proliferation in the absence of MYC/MYCN deregulation and counteract the effect of mTOR inhibition. In contrast, KNS42 and SF188 were able to maintain the same proliferation rates, suggesting that these cells have adapted to the hypoxia-induced effects on MYC/MYCN through AKT and ERK reactivations or diverse genomic alterations like the CDK4 and CCND2 gene amplifications seen in SF188. Indeed, the specific inhibition of HIF-1α or HIF-2α by siRNA interference had no significant impact on proliferation in KNS42 or SF188, but the combined inhibition of both negatively did, confirming their alternative role to promote survival and proliferation [16, 20]. These results suggest that, in the context of a MYC/MYCN dysregulation, the unique inhibition of HIF-1α is not efficient to control cell proliferation, whereas HIF-2α targeting seems to be efficient in MYC amplified cells as already described in other cancers [51]. It also suggests that HIF-2α can modulate proliferation even at low levels of expression, but it may depend on a complex transcriptional synergy with HIF-1α [15, 19, 21, 51]. Interestingly, in absence of MYC/MYCN deregulation, in UW479, the inhibition of HIF-1α and HIF-2α was sufficient to inhibit proliferation, confirming their role as drivers of tumor cell survival and might reverse the potential resistance to concomitant mTor/HIF-1α inhibition in case of highly HIF-2α over-expression.

To go further, helping to describe precise profiles linking genomics and metabolomics to treatment response [52], we performed such combined analyses in those *in vitro* models to establish distinct profiles of response to rapamycin and irinotecan. While SF188 and UW479 were relatively sensitive to rapamycin, KNS42 was resistant. Similarly to the hypoxia-induced mTOR inhibition, the pharmacological inhibition of mTOR by rapamycin led to a strong feed-back activation of mTORC2 and AKT in KNS42. As glycolysis, glutaminolysis and lipogenesis processes were not inhibited by small doses of rapamycin and even promoted under hypoxia in KNS42 despite a strong inhibition of HIF-1α accumulation; it is likely that HIF-2α and AKT might be drivers of resistance to rapamycin in this cell line. A recent publication also described MYCN as a regulator of cell metabolic adaptation independently from HIF-1α throughout the inhibition of NDRG family [53]. In fact, NDRG1 is repressed under hypoxia in KNS42 and activated by HIF-1α induction in SF188 and UW479. These results are correlated with the modest anti-proliferative effects obtained with siRNAs against HIF-1 in KNS42. In SF188, the modest activity of rapamycin could be related to ERK activation and the subsequent less inhibition of mTOR. A strong anti-glutaminolytic effect was associated with rapamycin treatment response in SF188. The sensitivity to rapamycin was also correlated with the accumulations of aspartate and glycine, previously described and correlated to a decrease of NTP/dNTP synthesis [54]. An increase of free amino acids was also observed in the rapamycin sensitive cell lines and could be related to a global inhibition of protein synthesis and/or an increase of autophagy due to mTOR inhibition [54].

The PCho:GPC ratio was the best to recapitulate the sensitivity to irinotecan. Its increase was related to high proliferation rates and invasiveness in glioma tumors, as in UW479, whereas the diminution of this ratio was correlated with response to therapy and considered as a surrogate marker for growth inhibition, as observed in SF188 and KNS42 sensitive cell lines [55]. Irinotecan, known as a TOP1 inhibitor, was also reported to inhibit HIF-1α gene transcription [35, 36, 56]. In our cell lines, irinotecan seemed to have only modest effects on HIF-1α expression. Nevertheless, a synergy could be evidenced with the combination of both drugs in the three cell lines, even at low doses. Interestingly, this complex synergy was significantly recapitulated by metabolomic changes, but also by protein or transcriptomic variations and confirmed the increased efficacy especially in SF188. The most complete response was traduced in SF188 by concomitant glutaminolysis, amino-acid and lipid synthesis inhibitions.

Most patient tumors seem to present an over-expression of either AKT or ERK, when analyzed by immunohistochemistry. The association with a MYC amplification was present in one third of the population recapitulating closely the SF188 protein profile. In addition, p-AKT/mTor was evidenced in another third and assimilated to KNS42 profile. Only 3 tumors had a HIF-2α over-expression without an alteration in p-ERK/mTor pathway and could be considered as potential resistant patients to our combination. The remaining tumors are characterized by a transitional profile described only in the sensitive cell lines. Finally, as could be expected in their protein signature, the patient derived cell line, TC68 showed a good sensitivity to rapamycin plus irinotecan treatments, as well as in SF188 cells, and TC35 presented an intermediate response to the combination as well as in KNS42 cell line. Despite the limitations of the *in vitro* models, these genotypes seem to be associated with specific intrinsic responses, which should be taken into consideration for resistance prediction and patient treatment.

In conclusion, using an *in vitro* model of hypoxia, susceptible to mimic more closely the *in vivo* hypoxic tumor conditions, we depicted metabolomic profiles of response to the combination of rapamycin plus irinotecan, determined by a balance between upstream signals in MYC and mTOR/HIF-1/2α pathways. Hence, we confirmed the high chemo-resistant phenotype driven by hypoxia through the induction especially of HIF-2α. We also showed that in the presence of MYC or NMYC dysregulation, the unique inhibition of HIF-1α was not sufficient and necessitated a combined approach. These profiles, which can be easily identified by IHC and MRI spectroscopy could be finally used as predictive biomarkers in future patients treated with the combined therapy.

## MATERIALS AND METHODS

### Patients

20 pediatric patients with supratentorial pHGGs and 6 with DIPG were included in the study after obtaining written informed consents. Aged from 8 to 19 years, they were treated between January 2005 and December 2013 in Strasbourg's University Hospital. The research protocol was validated by the local institutional ethic committee for human tissue experiments. The anonymized fresh-frozen or paraffin-embedded tumor samples were collected at diagnosis from surgical biopsy or resection and stored at the Biological Resource Centre (CRB) of Strasbourg (declaration number DC-2013-1826, May 2013).

### Cell lines and reagents

Three cell lines, SF188, KNS42 and UW479, were already established and tested in multiple assays. TC68 is a new tumor-derived cell lines obtained from a DIPG tumor with specific molecular characteristics described in Figure 1B (this tumor is marked in the histone mutation raw with a bar). A second primary cell line TC35 was also developed from a thalamic grade 4 glioma with a histone K27M mutation (this tumor is marked with a star on the histone mutation raw in the same Figure 1B).

Cell lines were cultured in DMEM/F-12 GlutaMAX™ (Gibco^®^) with 10% FBS. TC68, as well as KNS42 and SF188, grew as neurospheres with a FBS-free medium supplemented with B27 (Gibco^®^), FGF (Millipore^®^) and EGF (Gibco^®^). TC35, as well as UW479, are adherent cell lines. All cells were maintained at 37°C in normoxic (21% O_2_) or hypoxic (94% N_2_, 5% CO_2_, 1% O_2_) conditions in a Sanyo® incubator. Rapamycin was obtained from LC Laboratories (Woburn, USA) and irinotecan from Sandoz (Levallois-Perret, France). For hypoxic analyses, a prior progressive adaptation to hypoxia was realized to reach a final 1% concentration.

### Proliferation and growth inhibition assay

Proliferation and growth inhibition were determined using a crystal violet staining viability assay with optical spectrophotometry analysis as previously described [57]. For each drug, the GI50 (dose leading to 50% of the maximal growth inhibition effect on cultured cells) was assessed at 72 hours after treatment exposure. Values of optical density are reported as means+/−SD (standard deviation) obtained from three independent experiments.

### Protein analyses

#### Antibodies

For immunohistochemical, immunocytological and/or immunoblotting assays, the following antibodies were used against: αβ tubulin, LC3B, phospho-p44/42 MAPK (ERK1/2) (Thr 202, Tyr 204), phospho-RICTOR (Thr 1135), phospho-S6RP (Ser 235) and purchased from Cell Signaling Technology (Ozyme, St-Quentin-en-Yvelines, France). GLUT1, HIF-1α, HIF-2α, PARP, cleaved PARP, phospho-STAT3 (Tyr705) were purchased from Abcam (Paris, France), and phospho-H2AX (Ser 139) from Merck Millipore (Darmstadt, Germany). The histone status was assessed by immunohistochemical staining with an antibody detecting the loss of nuclear expression of the trimethylated lysine 27 (H3K27me3) in histone H3 (Diagenode, Belgium).

### Western blot analyses

Proteins were extracted after 24h of drug exposure and processed for western blotting as previously described [58]. Quantification of protein amounts was performed comparatively to alpha beta tubulin expression with ImageJ software (http://imagej.nih.gov). These measures were then normalized according to the control sample and expressed as means +/− SD obtained from replicates of two independent experiments.

### Immunohistochemistry (IHC) and immunocytofluorescence

FFPE tumor samples from patients were gathered on a tissue-microarray (TMA) block using a tissue arrayer (MTA BOOSTER 01 V2.04, Excilone, France) with 3 representative areas selected per tumor. IHC was performed using an automated tissue staining system (Ventana Medical Systems, Inc., Tucson, AZ, USA). Two pathologists analyzed protein expression independently. The scoring system was combining the degree of positivity and the proportion of positive cells. Indeed, more than 50% positive tumor cells and a moderate to strong intensity were defining an over-expressed protein in our samples. PTEN analysis, as well as H3K27m3 staining, was considering as a loss, when no protein expression was detected in tumor cells on the slides. The cell lines were cultured on Lab-Tek Chamber Slide™ Systems (Thermo Fisher Scientific, Waltham, USA) until 60% confluence and processed for immunocytofluorescence staining as previously described [35, 36, 57].

### RNA analyses

#### siRNA transfection

Pre-designed small interfering RNAs (siRNA) duplexes against HIF-1α and HIF-2α were purchased from Qiagen (Courtaboeuf, France). Cells were transfected using Lipofectamine RNAiMAX (Invitrogen, Life Technologies, St Aubin, France) according to the manufacturer's instructions. Two different siRNAs were tested alone or in combination for each target, as well as a scrambled sequence control duplex. The efficacy of transfection was confirmed with RT-PCR on RNAs extracts and immunoblotting on protein extracts made at 48 h post-transfection. The effect on proliferation was determined using crystal violet viability assay at 24, 48 and 72 h post-transfection.

#### mRNA expression profiling analyses

Expression profiling of 5 pHGGs, compared to 10 pilocytic astrocytomas and 4 grade 1 gliomas, was performed. Those 2 DIPGs, 2 thalamic and 1 hemispheric pHGG are including in our whole cohort of 26 patients. The cell lines (KNS42, SF188 and UW479) were analyzed in normoxia and hypoxia conditions at different treatment checkpoints: before any treatment, after 24h treatment exposure with rapamycin (10 nM), irinotecan (5 μM) and both drugs. All those experiments were performed in duplicate. Gene expression analysis was performed using Affymetrix U133 Plus 2.0 chips. Data were read with the affy package and normalized with rma. Gene expression was annotated with ensembl genes from build 37 assembly 71.

### DNA analyses of semi-quantitative PCRs (qPCRs) and gene sequencing

*H3F3A, HIST1H3B, PIK3CA*, *IDH1*, *BRAF* and *PDGFRA* genes were directly sequenced with a standard Sanger technique. Microsatellite analyses were performed for *PTEN* and *TP53* genes. qPCRs were performed for the determination of copy number alterations of *PDFGRA* and *MET* comparatively to *APP* and *DCK* reference genes. All these techniques were performed as previously described [58, 59].

### HRMAS NMR spectroscopy

HRMAS NMR spectroscopy was performed for KNS42, SF188 and UW479 in normoxic and hypoxic conditions of proliferation and after 24h exposure with rapamycin (10 nM), irinotecan (5 μM) and both drugs. Six replicates for each condition were analyzed. For cell lines, a pellet of 10^7^ cells was collected and directly snap-frozen in liquid nitrogen. HRMAS NMR spectra were analyzed on a Bruker Avance III 500 spectrometer operating at a proton frequency of 500.13MHz. HRMAS NMR data acquisition and spectra processing were performed as previously described [60, 61]. A one-dimensional (1D) proton spectrum was acquired for each sample. The chemical shift was referenced to the peak of the methyl proton of L-lactate at 1.33 ppm. Two-dimensional (2D) homonuclear and heteronuclear experiments were also recorded in order to confirm resonance assignments. The quantification procedure was based on the pulse length-based concentration measurement (PULCON). Spectra were normalized according to each sample weight and calibrated using the signal intensity of a 19.3 nmol reference solution of lactate. Quantification results were expressed as nmol/mg of tissue. Metabolites were assigned using standard metabolite chemical shift tables [60–62].

### Statistical analyses

GI50 and combination indexes (CI) were determined according to the Chou & Talalay method, using the Calcusyn software (Biosoft®, Cambridge, UK). A CI greater than 1.2 means was considered as a drug antagonism, a CI less than 0.8 as a drug synergism and, when it was comprised between 0.8 and 1.2, it was concluded as an additive effect.

For mRNA expression profiling, a significant differential expression was assigned as a 2 fold up and a 2 fold down in comparison with control. Up and down-regulated genes were determined for each drug versus a control (untreated cell lines) in normoxic and hypoxic conditions. Comparisons were performed between hypoxic and normoxic controls for each cell line, but also between each treatment condition (irinotecan, rapamycin and combination of rapamycin plus irinotecan). Genomic data were clustered as a whole and separately for each cell line, using standard un-supervised and supervised analyses. Gene-set enrichment analyses (GSEA) were performed for detection of activated pathways.

A multivariate network based method, the Algorithm to Determine Expected Metabolite Level Alterations Using Mutual Information (ADEMA), was used to assess the changes in metabolite concentrations of NMR HRMAS spectroscopy analyses. The algorithm was predicting the changes in levels of metabolites between the cases and the control using mutual information. We determined the relevant metabolites and grouped them using the metabolic network topology provided in Kyoto Encyclopedia of Genes and Genomes (KEGG) [62, 63] and the metabolic atlas by Selway et al. [64]. We used 6 discrete metabolite levels and probabilistically assigned an observation to at most 4 levels. We ran ADEMA 18 times with the 6 tested cases comparing the 3 cell lines (SF188, KNS42 and UW479) in two conditions of culture (hypoxia and normoxia). The metabolic profiles of the controls were compared to the metabolic profiles of the cells treated with irinotecan, rapamycin and the combination of both drugs in both normoxic and hypoxic conditions.

## SUPPLEMENTARY FIGURES


