# Supplementary material for: Characterization of the transcriptional and metabolic responses of pediatric high grade gliomas to mTOR-HIF-1α axis inhibition
Source: Oncotarget. 2017 Mar 23;8(42):71597–617. doi: 10.18632/oncotarget.16500 (PMC5641075; doi:10.18632/oncotarget.16500)
Supplement: Supplementary file 1 [file oncotarget-08-71597-s001.pdf]

Characterization of the transcriptional and metabolic responses of pediatric high grade gliomas to mTOR-HIF-1α axis inhibition

SUPPLEMENTARY FIGURES

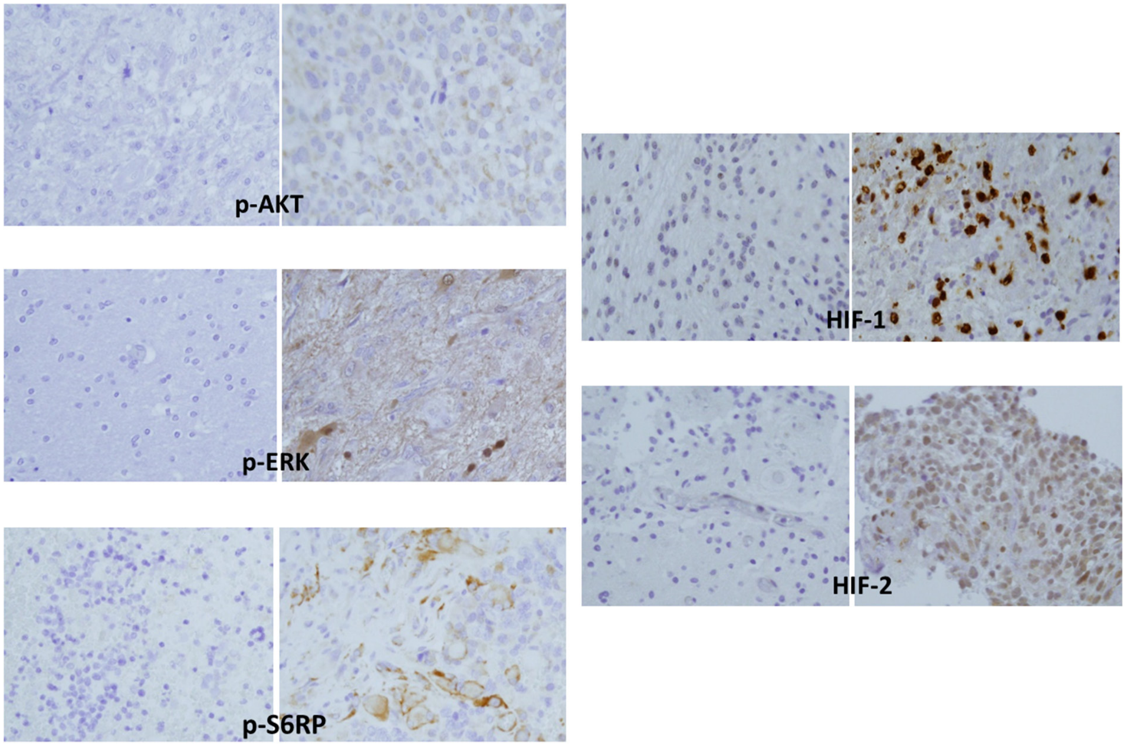

Supplementary Data 1: Immunohistochemical analyses on tumor slides exploring mTor/HIF-1α pathway. An example of negative result (left side) and paired positive staining (right side) is showed for each biomarker involved in mTor/HIF-1α pathway (e.g. p-AKT, p-ERK, p-S6RP, HIF-1α and HIF-2α) (magnification x40).

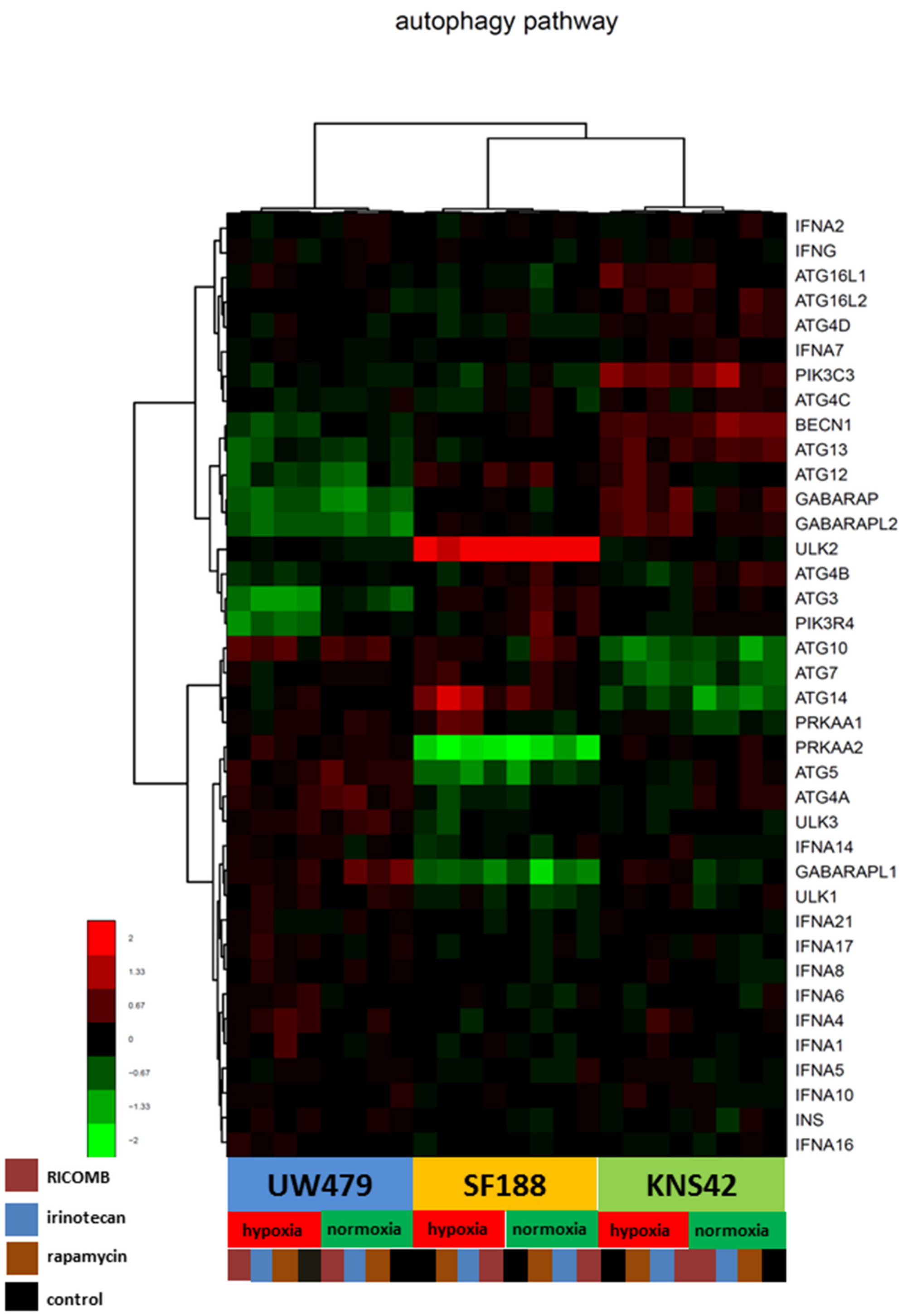

Supplementary Data 2: Effects of rapamycin, irinotecan and the combination of both (RICOMB) on autophagy pathway in KNS42, SF188 and UW479.
